# Rule Changes to Increase Shared Medal Winning at the Olympics

**DOI:** 10.3389/fspor.2022.885640

**Published:** 2022-04-26

**Authors:** Feifei Li, Will G. Hopkins

**Affiliations:** ^1^Centre for Health and Exercise Science Research, Department of Sport, Physical Education and Health, Hong Kong Baptist University, Hong Kong, China; ^2^College of Sport and Exercise Science, Institute for Health and Sport, Victoria University, Melbourne, VIC, Australia

**Keywords:** athletic performance, fair play, medal sharing, Olympics, tie

## Abstract

One of the most inspirational moments of the Tokyo Olympics was the sharing of the gold medal in the men's high jump. Rule changes that allow more medal sharing when athletes and teams are effectively equal in ability would improve the entertainment value of the Olympics, reward more athletes for their years of dedication to sport, and augment the Olympic ideal of fair play. Medals in all events are decided by a time, distance or points score in a final. When scores differ by ~0.1 or less of the variability in the score between competitions, the athlete or team with the better score would obtain a better score on average in only 52% of subsequent competitions, representing medals determined effectively by a coin toss. We have therefore quantified the medal sharing at the Tokyo Olympics that would have occurred if medals had been shared with such score differences (converted to rounded times or distances separating athletes in a final) in events with known variability between competitions (canoeing, kayaking, rowing, swimming, track and field events). In these events, 10%, 14% and 14% respectively of gold, silver and bronze medals would have been shared. The men's high jump would have produced three golds. Most of the sharing (68%) would have occurred with male athletes, presumably because greater depth of competition with males results in smaller differences between athletes at the highest level. The variability of performance scores in other events between competitions would need researching to establish maximum score differences for medal sharing in these events. For all events, the rule changes should exclude counting back, penalty shoot-outs, tie-breakers and any other methods for avoiding ties in the final. The acceptability of these rule changes to athletes, coaches and spectators (for example, in terms of separation of the athletes at the finishing line) would also need to be investigated.

## Introduction

An unforgettable moment in the Tokyo Olympics was the sharing of the gold medal in men's high jump. Three athletes reached a height of 2.37 m, but on count-back of successful prior jumps, one was awarded the bronze, while the other two agreed to share the gold. Most commentators regarded the outcome as inspirational and in keeping with the Olympic spirit (e.g., Gregory, 2021).

**Figure 1:**
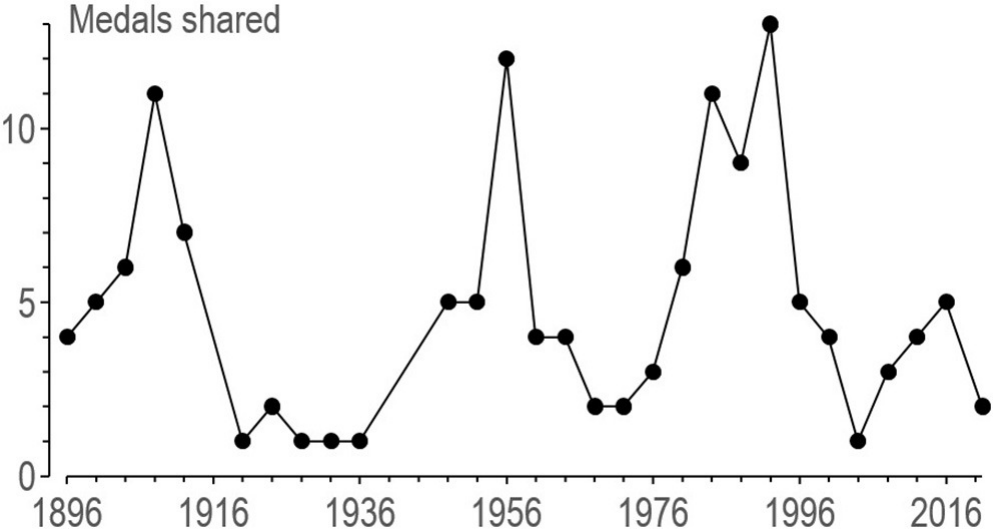
Count of shared medals arising from ties in the Summer Olympics. Data are from Wikipedia (2021). Thirteen three-way ties were each counted as two instances of medal sharing, and one four-way tie was counted as three instances.

There has been at least one shared medal in every Summer Olympic Games (Figure 1). The maximum of 11 occurred at Barcelona in 1992, and in recent Olympics the average is around three, which is only about 0.3% of the ~1,000 medals on offer. Indeed, the rules for each sport are written apparently to avoid ties: a count-back followed if necessary by a jump-off in the high jump, a penalty shoot-out in soccer, a tie-breaker in the fifth set in tennis, and so on. The sharing of the gold medal was in accordance with the rules, which allow the athletes to forego the jump-off (World Athletics, 2019).

Torres and McLaughlin (2003) provided a review of the philosophical literature on ties in sport and made the following compelling defense of ties.

When relative abilities in an athletic contest are so similar that they do not warrant distinguishing a winner and a loser, forcing this distinction [with a tie-breaker] would produce a result not merited by the performance [ability] of the contestants… The comparative purpose of competitive sport is fully served by ties; a comparison does not need to end in the establishment of superiority or inferiority. In sport, as in life, people might deem it perfectly acceptable that two things or phenomena are similar in relevant ways after careful evaluation.

We agree, and we therefore believe that the rules for each event at the Olympics should be changed to allow more medal sharing, not only when the athletes' or teams' final scores are equal, but also when any difference between the scores is negligible. More medal sharing would reward more athletes for their years of dedication to sport, reward more supporters, organizations and countries they represent, result in more medal-dependent investment in sports, improve the entertainment value of the Olympics, and augment the Olympic ideal of fair play. In this study we have quantified the medal sharing that would have occurred at the Tokyo Olympics in a range of events for which a negligible difference in performance could be estimated and justified.

Medals in all events are decided by a performance time, distance, or points score in a final. If the final were repeated, an athlete or team would seldom have exactly the same score, owing to irreducible random variability in performance between competitions (Hopkins et al., 1999). An athlete or team who beats another by a margin sufficiently greater than the variability would almost always beat that athlete in future competitions, and the winner would therefore deservedly earn the medal ahead of the other athlete or team. Conversely, if the margin were small enough, the winner would have only a negligible advantage in terms of beating the other athlete or team in future competitions, and the medal should therefore be shared. What margin would produce a negligible advantage? We have addressed this question by considering the sampling distribution of the difference score between the two athletes (or teams) in future competitions.

## Methods

The uncertainty in the difference score *d* of the athletes in a first competition is a standard error of *s*√2, where *s* is the standard deviation representing the variability of each athlete's performance. In future competitions, this standard error combines with the standard deviation of the difference score between the athletes, again *s*√2, to give a standard error for the sampling distribution of √[(*s*√2)^2^ + (*s*√2)^2^] = 2*s*. The difference *d* is therefore a *z* score of *d*/(2*s*), and the probability that the first athlete will beat the other athlete in future competitions is given by the cumulative normal distribution, NORMSDIST(*z*) or NORM.S.DIST(*z*,1) in Excel. If *d* = 0.25*s*, the probability is 0.55 or 55%; in other words, for every 10 future competitions, the first athlete would beat the second athlete 5.5 times, the second athlete would beat the first 4.5 times, so the first athlete would beat the second in one extra competition every 10 competitions. Winning an extra medal every 10 competitions for a top athlete is regarded as the smallest important enhancement of performance (Hopkins et al., 1999), so we could in principle set the sharing margin to 0.25*s*. With a smaller margin, *d* = 0.1*s*, the first athlete would beat the second in only 52% of future competitions, outcomes effectively the same as the 50% provided by tossing a fair coin. We have chosen a margin of 0.1*s* rather than 0.25*s* for two reasons. First, the published estimates of *s* (Malcata and Hopkins, 2014) have come from within-season variability and therefore potentially include real changes in an athlete's performance (i.e., changes additional to the random changes that would occur if the competition were re-run within days rather than weeks or months). Secondly, margins of 0.25*s* could translate into distances perceived as unacceptably large between throws or jumps in field events and between athletes at the finishing line in track and other timed events.

The Olympic events chosen were those in which athletes compete as individuals for a best time or distance and for which the variability of performance of top athletes between competitions has been reasonably well-quantified (Malcata and Hopkins, 2014). Track athletics, field athletics, swimming, rowing, kayak and canoe events (sprint and slalom) were selected for further analysis. The variability is ~1% for performance in track running, swimming, and rowing, 1–2% for kayaking and canoeing, and 1–4% for field events. Margins for sharing a medal were chosen as a “rounded” difference in the distance (field events) or time (all other events) that represented ~0.1 or less of the variability in performance in the given event. Olympic-final distances and times were obtained from BBC Sport (2021) and Olympics (2021) websites for the top place-getters. Differences between consecutive place-getters were calculated, and any differences equal to or less than the chosen margin were counted as shared medals.

For track running and swimming events, the medals are awarded on the basis of times recorded to 0.001 s, but the times are published only to the nearest 0.01 s. Where the published difference was equal to our chosen margin, we calculated that the difference recorded to 0.001 s would be less than or equal to our margin only for 0.55 of such differences, so these differences contributed 0.55 to the count of shared medals. There were two such occurrences in swimming and a similar occurrence in the men's marathon, where the times are recorded to 0.1 s but published only to the nearest second.

## Results

Table 1 shows our chosen distance margins for sharing medals in the Olympic field events, with the distances of the first four place-getters in each event and the distances that would have resulting in medal sharing. The gold was already shared between the first two place-getters in the men's high jump, but with an equal best jump, the third placed athlete would also share the gold. Totals of six and five instances of medal sharing would have occurred in the men's and women's field events, respectively.

**Table 1:** Variability of performance in field events, suggested margins for medal sharing derived from the variability, and performance distances of the first four place-getters at the Tokyo Olympics.

	**Variability^a^**	**Margin**		**Distance (m)**			
	**(%)**	**(m)**	**(%)**	**1st**	**2nd**	**3rd**	**4th**
**Men's**							
High jump	(1.6)	0.01	0.42^b^	2.37	2.37	2.37	2.35
Pole vault	1.9	0.01	0.17	6.02	5.97	5.87	5.80
Long jump	2.3	0.01	0.12	8.41	8.41	8.21	8.18
Triple jump	3.1	0.05	0.28	17.98	17.57	17.47	17.44
Shot put	(2.6)	0.05	0.21	23.30	22.65	22.47	21.88
Discus throw	1.0	0.05	0.07	68.90	67.39	67.07	67.02
Hammer throw	(1.0)	0.1	0.12	82.52	81.58	81.53	80.39
Javelin throw	4.7	0.1	0.11^c^	87.58	86.67	85.44	85.30
**Women's**							
High jump	1.6	0.01	0.49^b^	2.04	2.02	2.00	1.98
Pole vault	(1.9)	0.01	0.20	4.90	4.85	4.85	4.80
Long jump	(2.3)	0.01	0.14	7.00	6.97	6.97	6.91
Triple jump	1.8	0.03	0.19	15.67	15.01	14.87	14.84
Shot put	2.6	0.05	0.24	20.58	19.79	19.62	19.57
Discus throw	(1.0)	0.05	0.07	68.98	66.86	65.72	65.01
Hammer throw	(1.0)	0.1	0.13	78.48	77.03	75.49	74.41
Javelin throw	(4.7)	0.1	0.15^c^	66.34	64.61	64.56	64.00

a *Variability data are standard deviations from Malcata and Hopkins (2014). Values in parentheses are estimates assumed to be the same as for the opposite gender*.

b *High-jump margin is somewhat >0.1 of the variability, because height of the bar is set in whole centimeters*.

c *Javelin margin is somewhat < 0.1 of the variability, on the assumption that a margin >0.1 m (10 cm) would be unacceptable*.

*Rectangles enclose distances that would have resulted in a shared medal, with the color of the medal determined by the better place-getter (1st = gold, 2nd = silver, 3rd = bronze)*.

For the track, walking and marathon events in Table 2, our chosen time margins for sharing medals are also shown expressed as distances representing the maximum separation of athletes at the finishing line that would result in medal sharing. The smallest separations (~10 cm) are in the shortest sprint and hurdles events, while the largest separations (~7–10 m), are in the race walks and marathon. There would have been six occurrences of medal-sharing in the men's events, but none in the women's.

**Table 2:** Suggested margins for medal sharing derived from the variability of performance in track, walking and marathon events (standard deviations of ~1%: Malcata and Hopkins, 2014), and performance times of the first four place-getters at the Tokyo Olympics.

	**Margin**			**Time (s)**			
	**(s)**	**(%)^a^**	**(cm)**	**1st**	**2nd**	**3rd**	**4th**
**Men's**							
100 m	0.01	0.10	10	9.80	9.84	9.89	9.93
110-m hurdles	0.02	0.15	17	13.04	13.09	13.10	13.14
200 m	0.02	0.10	20	19.62	19.68	19.74	19.93
400 m	0.04	0.09	36	43.85	44.08	44.19	44.21
400-m hurdles	0.02	0.04	17	45.94	46.17	46.72	47.08
800 m	0.05	0.05	38	105.06	105.23	105.39	105.92
1500 m	0.05	0.02	36	208.32	209.01	209.05	209.56
3,000-m steeplechase	0.1	0.02	61	488.90	490.40	491.50	495.00
5,000 m	0.2	0.03	129	778.15	778.61	779.05	779.17
10,000 m	0.5	0.03	301	1663.22	1663.63	1663.88	1666.16
20-km race walk	2	0.04	822	4,865	4,874	4,888	4,906
50-km race walk	2	0.01	724	13,808	13,844	13,859	13,868
Marathon	2	0.03	1,086	7,718	7,798	7,800	7,802
**Women's**							
100 m	0.01	0.09	9	10.61	10.74	10.76	10.91
100-m hurdles	0.01	0.08	8	12.37	12.52	12.55	12.60
200 m	0.02	0.09	19	21.53	21.81	21.87	21.94
400 m	0.04	0.08	33	48.36	49.20	49.46	49.61
400-m hurdles	0.05	0.10	39	51.46	51.58	52.03	53.08
800 m	0.05	0.04	35	115.21	115.88	116.81	116.90
1500 m	0.05	0.02	32	233.11	234.50	235.86	237.60
3,000-m steeplechase	0.1	0.02	55	541.45	544.79	545.39	546.16
5,000 m	0.2	0.02	114	876.79	878.36	878.87	879.62
10,000 m	0.4	0.02	222	1795.32	1796.18	1801.72	1824.27
20-km race walk	2	0.04	747	5,352	5,377	5,397	5,405
Marathon	2	0.02	948	8,840	8,856	8,866	8,918

a *Margins for distances >400 m are somewhat < 0.1%, on the assumption that the distance margins at the finish line would be otherwise unacceptable*.

*Rectangles enclose times that would have resulted in a shared medal, with the color of the medal determined by the better place-getter (1st = gold, 2nd = silver, 3rd = bronze)*.

Time margins chosen for the swimming events ranged from 0.02 s for 50-m freestyle through 1.0 s for the marathon; the corresponding distance margins were 4 cm through 1.5 m. There would have been 6.1 instances of sharing in the men's swimming and 2.5 in the women's. In rowing, a sharing margin of 0.2 s for all men's and women's events would produce distance margins of 0.9–1.2 m; five instances of sharing would have occurred in the men's events, and two in the women's. In kayaking and canoeing, time margins ranged from 0.02 s for the 200-m sprint events through 0.2 s for the 1,000-m events; corresponding distance margins were ~10 cm through ~1.0 m. Distance margins were not estimated for the slalom events, because these were competed individually as time trials. There would have been three occurrences of sharing in men's kayaking and canoeing, and two in the women's.

We analyzed 48 male and 48 female events in total, representing 96 each of gold, silver and bronze medals that could be shared; of these, 10%, 14% and 14% respectively would have been shared with our sharing margins. Most of the sharing (68%) would have occurred with male athletes.

## Discussion

Although the sample of events is small, a larger sample would probably show the same pattern of more medal sharing with silver and bronze than with gold. This pattern would be a consequence of the distribution of athlete abilities in any given event: medals are decided by performances on the tail of the distribution, the tail has an exponential shape, and therefore on average there are larger differences between athletes further out on the tail. There would also likely be more sharing in male than in female events, presumably because a greater depth of competition with males would result in smaller differences between top athletes (Schorer et al., 2015). Sharing amongst female athletes could be brought to a level similar to that of males by increasing the sharing thresholds in relevant events, especially if females were more supportive of medal sharing.

The use of sharing margins would produce an interesting scenario, when the score differences between consecutive place-getters are all just a little less than the margin. The first two athletes would share the gold medal, and normally the third athlete would receive the bronze. However, when the difference between the first two athletes is greater than the sharing margin, the second and third athlete would share a silver. In the spirit of increasing the number and color of medals awarded to athletes in recognition of their ability and dedication, the third athlete could be awarded a silver when the first two athletes share the gold. The fourth athlete would share the silver with the third athlete, if the score difference between third and fourth athletes was also just less than the sharing margin, which would then allow for a bronze to be awarded to the fifth athlete and possibly shared with the sixth athlete. Of course, this extreme scenario is most unlikely to ever occur, but elevating the third place-getter to a silver and awarding also a bronze (or two) would occur sometimes.

Is the medal-sharing we propose consistent with the Olympic ideal of fair play? The Olympics site mentions the term *fair play* in connection with *respect* toward rules, opponents and oneself (International Olympic Committee, 2021), but there is no mention of what should constitute fair play in resolving ties. We therefore have to ask: is it fair play when the outcome of many years of dedication to a sport is rewarded with a medal decided by the toss of a coin? Surely not. And is it fair play when the athlete or team who thereby “wins” a gold medal is rewarded with greater recognition and adulation than the athlete or team who ends up with the silver? Surely not. It seems to us that the Olympic ideal of fair play would be enhanced by respect for the notion that athletes or teams with apparently equal ability on the basis of performance in a final should receive the same medal. The practical way to realize the medal-sharing we propose is to change the rules to allow for sharing margins and to remove rules about tie-breakers.

Medal sharing is also consistent with the Olympic motto *citius altius fortius*. The motto refers to athletes being better in their chosen sport, but there is nothing wrong with two athletes being equally better than others in the field. More medal sharing is also more consistent with the official motto revealed at the Beijing Winter Olympics: *together for a shared future* (Olympics, 2022).

The most important issue arising from our proposal concerns the acceptability of the sharing margins. The high jump provides a precedent for an existing margin (1 cm) that represents considerably more (0.42%) even than our generic margin of 0.1 of the variability of performance (0.1 of 1.6%). The existing margin is a consequence of the fact that the height of the bar is measured and set in whole centimeters, and the margin is evidently acceptable. Margins for distances and times in other events should therefore be acceptable in principle, but athletes and spectators might take some persuading that athletes visibly separated at the finishing line in timed events should share a medal. The most extreme case is the men's marathon, where a margin of 0.03%, which is already considerably less than our generic margin of 0.1% (0.1 of the variability of 1%), represents a separation of up to 10 m at the finishing line.

In conclusion, we have argued for medal sharing, when the margin between the athletes is sufficiently small that the outcome in repeated competitions would be decided by the toss of a coin (i.e., either athlete would be better than the other in ~50% of repeated competitions). We could have instead argued for not sharing only when the margin between the athletes is sufficiently large to give a high probability of the same outcome in repeated competitions (i.e., the better athlete in the first competition would be better in most repeated competitions). We have shown that the former approach would result in a modest increase in medal sharing. The latter approach would result in far more athletes winning medals, but it would be acceptable only if Homo sapiens were a much more caring and sharing animal than is manifestly the case in sport and in most other walks of life.

## Future Perspectives

The variability of performance scores between competitions in events other than those we have considered here would need researching to establish maximum score differences for medal sharing in these events. For all events, the rule changes should exclude counting back, tie-breakers, penalty shoot-outs, and any other methods for avoiding ties in the final. In some events, the rules for qualifications, heats, quarter- and semi-finals might need to be adjusted to avoid too many tied competitors reaching the final. In events with low point or goal scores, especially football, abolishing penalty shoot-outs might result in an unwieldy high frequency of tied matches. In our view, it would be better to change the event to produce higher scores (e.g., by increasing the frontal area of the goal) than to retain penalty shoot-outs, which do not identify teams that play a better preceding game. The acceptability of any rule or other changes would obviously need to be investigated with athletes, coaches, and spectators, especially in terms of separation of athletes at the finishing line.

## Data Availability Statement

The original contributions presented in the study are included in the article, further inquiries can be directed to the corresponding author/s.

## Author Contributions

All authors listed have made a substantial, direct, and intellectual contribution to the work and approved it for publication.

## Conflict of Interest

The authors declare that the research was conducted in the absence of any commercial or financial relationships that could be construed as a potential conflict of interest.

## Publisher's Note

All claims expressed in this article are solely those of the authors and do not necessarily represent those of their affiliated organizations, or those of the publisher, the editors and the reviewers. Any product that may be evaluated in this article, or claim that may be made by its manufacturer, is not guaranteed or endorsed by the publisher.
